# Early Changes of Mannose-Binding Lectin, H-Ficolin, and Procalcitonin in Patients with Febrile Neutropenia: A Prospective Observational Study

**DOI:** 10.4274/tjh.2014.0385

**Published:** 2016-12-01

**Authors:** Sibel Işlak Mutcal, Neşe Saltoğlu, İlker İnanç Balkan, Reşat Özaras, Mücahit Yemişen, Bilgül Mete, Fehmi Tabak, Ali Mert, Recep Öztürk, Şeniz Öngören, Zafer Başlar, Yıldız Aydın, Burhan Ferhanoğlu, Teoman Soysal

**Affiliations:** 1 İstanbul University Cerrahpaşa Faculty of Medicine, Department of Infectious Diseases and Clinical Microbiology, İstanbul, Turkey; 2 Medipol University Faculty of Medicine, Department of Internal Medicine, İstanbul, Turkey; 3 İstanbul University Cerrahpaşa Faculty of Medicine, Department of Hematology, İstanbul, Turkey; 4 Koç University Faculty of Medicine, Department of Internal Medicine, Division of Hematology, İstanbul, Turkey

**Keywords:** Febrile neutropenia, Infection, Mannose-binding lectin, H-ficolin, Procalcitonin, C-reactive protein

## Abstract

**Objective:**

The significance of mannose-binding lectin (MBL) and H-ficolin deficiency in febrile neutropenic (FN) patients and the correlation of these markers along with consecutive C-reactive protein (CRP) and procalcitonin (PCT) levels during the infectious process are investigated.

**Materials and Methods:**

Patients with any hematological malignancies who were defined to have “microbiologically confirmed infection”, “clinically documented infection”, or “fever of unknown origin” were included in this single-center prospective observational study. Serum levels of CRP, PCT, MBL, and H-ficolin were determined on 3 separate occasions: at baseline (between hospital admission and chemotherapy), at the onset of fever, and at the 72nd hour of fever.

**Results:**

Forty-six patients (54% male, mean age 41.7 years) with 61 separate episodes of FN were evaluated. Eleven patients (23.9%) had “microbiologically confirmed infection”, 17 (37%) had “clinically documented infection”, and 18 (39.1%) had “fever of unknown origin”. Fourteen (30.4%) patients had low (<500 ng/mL) initial MBL levels and 7 (15.21%) had low (<12,000 ng/mL) H-ficolin levels. Baseline MBL and H-ficolin levels did not significantly change on the first and third days of fever (p=0.076). Gram-negative bacteremia more frequently occurred in those with low initial MBL levels (p=0.006). PCT levels were significantly higher in those with microbiologically documented infections. Mean and median PCT levels were significantly higher in cases with bacteremia. There was no significant difference between hemoculture-positive and-negative patients in terms of CRP levels.

**Conclusion:**

Monitoring serum H-ficolin levels was shown to be of no benefit in terms of predicting severe infection. Low baseline MBL levels were correlated with high risk of gram-negative bacteremia; however, no significant correlation was shown in the follow-up. Close monitoring of PCT levels is warranted to provide more accurate and specific data while monitoring cases of bacteremia.

## Abstract (tr)

**Amaç:**

Febril nötropenik (FEN) hastalarda mannoz-bağlayıcı lektin (MBL) ve H-fikolin eksikliğinin önemi ve bu belirteçlerin enfeksiyon atağı sırasında ardışık C-reaktif protein (CRP) ve prokalsitonin (PCT) ölçümleri ile korelasyonu araştırılmıştır.

**Gereç ve Yöntemler:**

Bu tek merkezli prospektif gözlemsel çalışmaya, hematolojik malignite nedeniyle izlenen ve “mikrobiyolojik olarak doğrulanmış enfeksiyon”, “klinik olarak dökümante edilmiş enfeksiyon” veya “nedeni bilinmeyen ateş” tanıları konulan hastalar dahil edilmiştir. Serum CRP, PCT, MBL ve H-fikolin düzeyleri; başlangıçta (hastaneye başvuru ile kemoterapi başlangıcı arasında), ateş atağının başında ve 72. saatinde olmak üzere üç ayrı zamanda ölçülmüştür.

**Bulgular:**

Kırk altı (%54 erkek, ortalama yaş 41,7) hastada gelişen 61 ayrı FEN atağı değerlendirildi. Hastaların 11’inde (%23,9) “mikrobiyolojik doğrulanmış enfeksiyon”, 17’sinde (%37) “klinik dökümante enfeksiyon”, 18’inde (%39,1) ise nedeni bilinmeyen ateş mevcut idi. Başlangıç MBL düzeyi (<500 ng/mL) yedi hastada, H-fikolin düzeyi ise (<12,000 ng/mL) 14 hastada düşük bulundu. Bazal MBL ve H-fikolin düzeylerinin ateşin birinci ve üçüncü gününde anlamlı olarak değişmediği belirlendi (p=0,076). Başlangıç MBL düzeyi düşük olan hastalarda gram-negatif bakteremilerin daha sık ortaya çıktığı saptandı (p=0,006). PCT düzeyleri “mikrobiyolojik olarak doğrulanmış” enfeksiyonu bulunanlarda anlamlı olarak daha yüksekti. Medyan PCT düzeyleri tüm FEN epizodlarında anlamlı olarak yükselmiş bulundu.

**Sonuç:**

Ciddi bakteremilerin ön görülmesi açısından serum H-fikolin düzeylerinin izlenmesinin yararı olmadığı gösterildi. Düşük bazal MBL düzeyleri ile yüksek gram-negatif bakteremi riski arasında ilişkili olduğu belirlenmekle birlikte izlemde anlamlı korelasyon gösterilemedi. Bakteremi olgularının izleminde daha hızlı ve özgül veriler elde edebilmek için PCT düzeylerinin yakın izleminin gerekli olduğu sonucuna varıldı.

## INTRODUCTION

Blood stream infections (BSIs) due to invasive bacterial and fungal pathogens are major causes of infection related mortality. Gram-negative and gram-positive bacteremia account for 50%-60% of BSIs during febrile neutropenia (FN) episodes [1,2,3]. Nonspecific signs and symptoms and conventional microbiologic methods pose some problems in the diagnosis of severe infections in neutropenic patients. Hemoculture is still the standard diagnostic method, but the positivity rate is only about 20-50% in FN episodes [4] and microbial identification takes 2-6 days [1]. Definition of early diagnostic markers that will guide antimicrobial treatment is critical [5].

In current practice, antibacterial therapy is initiated immediately after blood cultures are obtained and before any other diagnostic procedures, in accordance with guidelines. Leukocytes and differential blood count, hemoglobin, platelets, serum glutamate oxaloacetate transaminase, serum glutamate pyruvate transaminase, lactate dehydrogenase, alkaline phosphatase, gamma glutamyltransferase, bilirubin, uric acid, creatinine, sodium, potassium, partial thromboplastin time, and C-reactive protein (CRP) are measured twice a week before and during therapy in the routine practice of our hematology section. Procalcitonin (PCT) is measured weekly throughout the neutropenic episode.

Mannose-binding lectin (MBL) is a plasma collectin (C-type lectin with a collagen-like domain) thought to have an important role in innate immunity [6]. Its lectin domain recognizes sugar patterns typical of microbial surfaces, while its collagen-like region facilitates microbial uptake by phagocytic cells. MBL can activate the complement by a mechanism similar to the classical pathway, but using MBL-associated serine proteases instead of C1r and C1s. The complement system provides immediate defense against infection and has proinflammatory effects. MBL deficiency is defined as a serum level of <500 ng/mL. It is a laboratory finding that does not necessarily equate to a clinical disorder. MBL deficiency is associated with a large and heterogeneous group of disease processes. However, subnormal levels are also found in healthy people. To date, there is no consensus on the clinical relevance of MBL deficiency or its treatment [7].

According to the results of the largest adult cohort, MBL deficiency is not correlated with more frequent or more prolonged febrile episodes during myelosuppressive chemotherapy in adults with hematological cancer, but severe infections are more frequent in MBL-deficient patients and first severe infection develops earlier in this group compared with nondeficient patients [8].

In this prospective study we aimed to confirm or refute these findings and to extend the investigation to one of the plasma ficolins, the Hakata antigen (H-ficolin). Ficolins share with collectins an overall quaternary structure resembling C1q and bind to bacteria and activate the complement using the lectin pathway of complement activation [9]. H-ficolin might therefore be a potentially useful marker of innate immunity. In this respect, the significance of MBL and H-ficolin deficiency in FN patients and the role of consecutive CRP and PCT measurements in the etiological differentiation of fever and in establishing a follow-up protocol are investigated.

## MATERIALS AND METHODS

The study was planned and conducted with a prospective methodology. All patients were consecutively evaluated and included in the relevant predefined case groups. Patients hospitalized in the hematology and hematopoietic stem cell transplantation units of the Cerrahpaşa Medical School Training Hospital with any hematological malignancies and who developed at least one episode of FN between February 2011 and July 2012 were included in the study. Patients were divided into 3 diagnostic groups as “microbiologically confirmed infection”, “clinically documented infection”, and “fever of unknown origin” according to German guidelines [10]. The patients were reevaluated at the end of the neutropenic episode and assigned to the relevant groups by the principle investigator, who was blinded to the laboratory results at that time.

### Study Protocol

Three separate blood samples were obtained from the patients on 3 separate occasions: at baseline (between hospital admission and chemotherapy), at the onset of fever, and 72 h after the first febrile spike. Empirical antipseudomonal antimicrobial treatment (piperacillin tazobactam or cefoperazone/sulbactam for the first episode and carbapenem for recurrent episodes or in case of increased risk of extended-spectrum beta-lactamase-producing gram-negative bacteria) was initiated in accordance with the FN guidelines [10] following hemoculture. Some patients with prolonged neutropenia developed more than one febrile episode and data were recorded separately for each.

### Data Collection

All required data were recorded on case follow-up forms from the first day of hospitalization. Demographic and clinical features including age, sex, comorbidities, vital signs, status of clinical sepsis, radiological data, microbiological data, antimicrobial treatment, and response data were recorded. Clinical and laboratory improvement within 96 h of treatment was defined as response to the antimicrobials.

### Inclusion Criteria

Patients with hematologic malignancies who developed an episode of FN were included. Neutropenia was defined as an absolute neutrophil count of ≤500/mm3 or 500-1000/mm3 but expected to fall below ≤500/mm3 within 24-48 h. Fever was defined as a single measurement of tympanic fever of ≥38 °C or at least 2 consecutive measurements of tympanic fever of ≥37.8 °C measured with 4 h intervals within 24 h of monitoring.

### Exclusion Criteria

Patients lacking any of the 3 blood samples during follow-up were excluded, along with those under 18 years or pregnant.

### Antimicrobial Treatment

There was no off-protocol intervention regarding antimicrobial use in FN episodes during the study period.

### Laboratory Analysis

Blood cultures were incubated for 7 days in an automated hemoculture system (BacT ALERT 3D, bioMérieux, France). Conventional biochemical methods and automated systems (API automation pour identification, bioMérieux) were used for identification.

Antimicrobial susceptibility tests were performed using the disk diffusion method in accordance with the relevant Clinical and Laboratory Standards Institute recommendations [11]. Blood samples were stored at -80 °C in accordance with the manufacturer’s recommendations (B.R.A.H.M.S., Hycult) and were tested after being thawed and centrifuged for 1 min. MBL, H-ficolin, and PCT levels were measured using Hycult MBL, enzyme-linked immunosorbent assay, and B.R.A.H.M.S. VIDAS methods, respectively.

### Statistical Analysis

SPSS 16.0 was used for statistical analyses. Categorical variables were analyzed with chi-square tests and continuous variables were analyzed with Student t or Mann-Whitney U tests. The Spearman correlation test was used to evaluate correlation between continuous variables. A p-value of <0.05 was accepted as statistically significant.

### Ethical Approval

This single-center, prospective, observational study was approved by the Institutional Review Board of Cerrahpaşa Medical School. All collected data were kept confidential.

## RESULTS

A total of 82 patients were registered. Sixty-one FN episodes in 46 patients were included in the study after excluding 36 patients lacking any of the 3 serum samples or not fulfilling the inclusion criteria. Twenty-five (54%) of the patients were male and the mean age was 41.7, ranging between 19 and 81 years. Distribution of diagnoses and number of FN episodes per diagnosis and patient are shown in Table 1.

The clinical manifestations of the cases with FN episodes are defined below:

1. Microbiologically + clinically documented infection: 11 cases (23.9%),

2. Only clinically documented infection: 17 cases (37%),

3. Fever of unknown origin: 18 cases (39.1%).

Eight (8/11) of the patients with microbiologically + clinically documented infection had primary bacteremia, 2 had bacteremia due to urinary tract infection, and 1 had urinary infection. Six of the pathogens isolated from blood cultures were gram-positive cocci and 4 were gram-negative bacilli. The most common gram-positive bacteria were methicillin-resistant coagulase negative staphylococci and the most common gram-negative bacterium was Escherichia coli.

Among those 17 cases with clinically documented infections, the source of infection was skin and soft tissue in 4, perianal abscess in 3, catheter exit site in 3, tooth abscess in 2, pneumonia in 1, myositis in 1, tracheostomy site in 1, surgical site in 1, and tonsillitis in 1. The rate of gram-negative bacteremia was significantly higher in cases with lower MBL levels when compared to cases with normal MBL levels (p=0.006) (Table 2).

**Figure 1:**
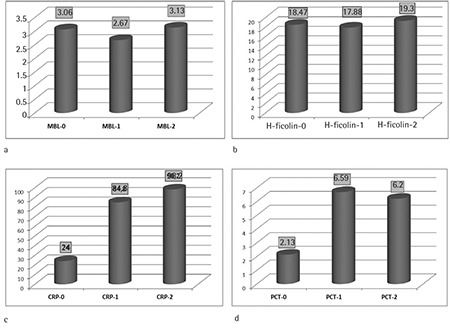
Serum mannose-binding lectin (a), H-ficolin (b), C-reactive protein (c), and procalcitonin (d) levels in patients. 0=at initial (between hospital admission and before chemotherapy), 1=at the onset of fever, and 2=at the 72nd hour of fever. MBL: Mannose-binding lectin, PCT: procalcitonin, CRP: C-reactive protein.

The average level of MBL was 3.060 ng/mL. Average levels of MBL did not significantly vary between the 3 measurements (MBL-0, MBL-1, and MBL-2) during the episodes of FN (p=0.076) (Figure 1a). Similarly, there was no significant difference between baseline, first day of fever, and third day of fever levels of H-ficolin (p>0.05) (Figure 1b). The average H-ficolin level of the cases was measured as 18.470 ng/mL. Median baseline CRP level (CRP-0) was measured as 24 mg/L (normal range: 0-5 mg/L). The average CRP level was elevated to 84.8 mg/L on the first day of FN episodes (CRP-1) and to 98 mg/L on the third day (CRP-2) (Figure 1c). This increase in the serial CRP levels was statistically significant (p<0.0001).

Median PCT levels (normal range: <0.5 ng/mL) were also significantly elevated in FN episodes (baseline PCT-0: 2.13 ng/mL, first day of fever PCT-1: 6.69 ng/mL, third day of fever PCT-2: 6.20 ng/mL; p<0.0001) (Figure 1d).

**Figure 2:**
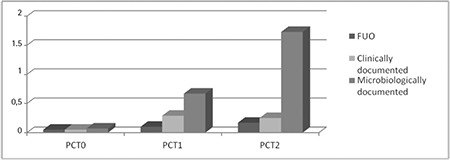
Median procalcitonin levels in febrile neutropenic patient subgroups. FUO: Fever of unknown origin, PCT: procalcitonin.

As shown in Table 3, PCT-1 was increased with borderline significance (p=0.055), while PCT-2 was significantly higher (p=0.028) when compared to baseline levels in cases with microbiologically documented infection. Kruskal-Wallis variance analysis revealed no significant difference in terms of CRP levels between predefined subgroups, while median PCT levels were significantly higher in those with microbiologically documented infections. Median PCT levels according to FN subgroups are shown in detail in Table 3 and Figure 2. The correlations between CRP, PCT, MBL, and H-ficolin levels during FN episodes were examined with Spearman correlation analysis. A strongly positive correlation was found between PCT-2 and CRP-2 values (p=0.008, r=0.39). CRP and PCT trajectories on the third day of fever were found to be parallel to each other. Similarly, there was a significant correlation between H-ficolin-2 and CRP-2 values (p=0.026, r=0.33).

While there was no significant difference between hemoculture-positive and hemoculture-negative patients in terms of CRP levels, mean and median PCT levels were significantly higher in cases with bacteremia (Table 3).

Mortality occurred in 9 (19.6%) of the 46 cases during the study period, 7 of which involved refractory hematologic malignancies and 2 bacteremia due to multiple-drug resistant gram-negative strains, one of which was carbapenemase-producing.

## DISCUSSION

Early diagnostic markers would ideally reflect the severity of the infection, help classify FN episodes as low-risk and high-risk in terms of likelihood of septic complications, and not be affected by the number of leukocytes and the course of underlying disease. CRP, as an acute phase marker and the most well-known biochemical marker of inflammation in patients with FN, was not found to be useful in the differential diagnosis of fever of unknown origin, bacteremia, and clinically documented infections in neutropenic patients. Similar to our study, CRP was found to be of no use in differential diagnosis in other studies [12,13,14]. False negativity that can be recognized in certain patient groups such as patients with leukemia, viral infections, systemic lupus erythematosus, progressive systemic sclerosis, dermatomyositis, ulcerative colitis, Sjögren’s syndrome, and cerebral infarction is an additional drawback for CRP as an acute phase marker [15].

Although the levels of serum PCT were determined to be lower in neutropenic patients when compared to those with intact immune systems, studies have shown that neutropenic patients had significantly higher PCT levels on days 0 and 2 in the case of sepsis [16]. The relationship between CRP and PCT levels during FN episodes was investigated in our study, and it was found that CRP is not a sensitive marker of early infection in neutropenic patients, while PCT would be preferred in the early diagnosis of sepsis. The rise of CRP or PCT from day 1 to day 3 in any patient group was evaluated as the expected peak serum levels related to the severity of infection rather than an ongoing uncontrolled sepsis. In our study, a slightly significant difference (p=0.055) was found between the first-day PCT levels (PCT-1) of FN episodes in bacteremic and nonbacteremic patients. Patients who had a microbiologically documented infection had significantly higher PCT levels on the third day (PCT-2) of the FN episode (p<0.05). CRP levels had no correlation with the clinical subcategories of FN episode, but higher PCT levels on the first day of fever (>0.5 ng/mL) and 72 h after the first peak of fever (with a cut point of >3-fold rise) were correlated with bacteremia, and particularly with gram-negative bacteremia. Although Svaldi et al. [17] reported that PCT levels did not significantly differ whether gram-negative or gram-positive bacteria were present when leukocyte count was <1.0x109/L, PCT levels were found to be higher in bacteremic patients than nonbacteremic patients and were more rapidly decreased in nondocumented infections in the studies of Akçay [13] and Secmeer et al. [18]. Nevertheless, de Bont et al. [19] reported similar levels of initial PCT levels at the onset of fever in bacteremic and nonbacteremic patients in a cohort of 66 patients. In the same study, cases with coagulase-negative staphylococci bacteremia were found not to have significant rises in PCT levels. Similar to our study, Fleischhack et al. [20] reported that children with FN infected with gram-negative bacteremia had higher PCT levels than did gram-positive cases [21].

It was concluded in a review published by Sakr et al. [21] of 30 studies that PCT levels were useful to distinguish the febrile episodes of systemic infection from noninfectious causes of fever. However, the capability to differentiate gram-negative and gram-positive bacteria in the etiology was limited.

MBL deficiency is defined as a serum level of <0.1 mg/L and was found in 5-10% of healthy adults. In a prospective study [21] evaluating 255 adult patients with hematologic malignancies and neutropenia, MBL levels were measured prior to the initiation of chemotherapy and on the first day of a febrile episode. MBL deficiency (<500 ng/mL) was detected in 62 (24%) of the patients.

The incidence of severe infection was higher among MBL-deficient patients than among non-MBL-deficient patients. In our study, MBL levels did not show any significant change in the first 3 days of FN. MBL levels were within normal ranges in 32 (69.5%) patients, 5 (15.1%) of whom had bacteremia due to gram-positive cocci. MBL levels were low (<500 ng/mL) in 14 (30.5%) of patients. Patients with low levels of MBL had a significantly higher rate of gram-negative bacteremia compared to patients with normal MBL levels (p=0.006), suggesting a correlation between MBL levels and risk of gram-negative infection.

In the review by Frakking et al. [22] investigating the correlation of infection in pediatric oncology patients with MBL deficiency and/or severity of infection, no relationship was found between low MBL levels and presence of sepsis, bacteremia, or fungal infection in 3 of the 5 studies, while the results of the other 2 studies were to the contrary. Although there are a variety of studies with different results in the literature [23,24,25,26,27], Peterslund et al. [28] showed a significant correlation between low levels of MBL and the development of bacteremia in adult patients with hematological malignancies.

In the study of Neth et al. [29] comparing 24 children with MBL levels of <1000 μg/L and 38 children with MBL levels of ≥1000 μg/mL, those with lower MBL levels developed FN episodes significantly more frequently. Schlapbach et al. [23] detected significantly more episodes of severe bacterial infections in patients with low MBL levels (<100 μg/mL), while those with higher MBL levels (>1000 μg/mL) had more frequent FN episodes due to microbiologically nondefined etiology. Kilpatrick et al. [24] demonstrated that patients with MBL of ≤0.1 mg/mL had significantly more major infections than no infections within the follow-up period (p<0.05). Deficiency of MBL (≤0.1 µg/mL) was significantly more frequent in patients with serious infections when compared to those with no infection within the follow-up period (p<0.05) [6] in a cohort of 128 patients with hematological malignancies treated by chemotherapy alone or combined with bone marrow transplantation. In the study of Bergmann et al. [25], no significant correlation between low MBL levels and the development of infection was detected in the FN episodes of patients with acute leukemia. Nevertheless, Horiuchi et al. [26] showed that low levels of a particular MBL genotype were related to severe bacterial infection. The dramatic differences reported in the studies of Peterslund et al. [28], Neth et al. [29], and Schlapbach et al. [23] in median MBL concentrations were virtually identical to those of the other patient categories. The studies of Klostergaard et al. [27], Frakking et al. [22], and Schlapbach et al. [23] revealed that infections due to gram-positive bacteria were more commonly observed in cases with low MBL levels.

MBL is one of the factors that may influence susceptibility to infection [6]. MBL variant alleles (implying low levels of circulating MBL) were found to be associated with major infections in recipients of allogeneic hemopoietic stem cell transplants [30]. Measuring the baseline MBL levels might be useful to define any predisposition to infections, particularly due to gram-negative bacteria, as a conclusion of our study. Baseline MBL levels might help categorize patients into high-risk and low-risk groups. A further study investigating how baseline MBL levels correlate with Multinational Association of Supportive Care in Cancer (MASCC) scores would be of great value. Given the easy treatment of its deficiency, baseline MBL will probably be a surrogate marker for the MASCC score, despite the current cost of the test.

H-ficolin was the other collectin investigated in our study. Seven patients had low levels of H-ficolin, 2 of whom developed gram-positive and 1 of whom developed gram-negative bacteremia. Due to the small number of patients in this group, no correlation was established between low H-ficolin levels and development of infection. In 4 of the 7 patients who had lower H-ficolin levels, MBL was also low. Two of these 4 patients had bacteremia.

In our study, MBL and H-ficolin levels did not show any significant variability in any subgroup of patients within the first 3 days of FN episodes. Different studies revealed different results owing to different patient groups, use of different chemotherapy regimens, and varying features of nosocomial causative agents within centers.

## CONCLUSION

Obtaining baseline MBL levels seems to be useful to predict severe infections, particularly due to gram-negative bacteria, in FN patients. Consecutive PCT levels are much more correlated with microbiologically documented infections, including bacteremia, and are preferable to CRP as a follow-up marker. No significant relation was found with baseline H-ficolin levels and risk of infection, and no significant change in serum level was detected during an emerging infection. Treatment of MBL deficiency would be a useful research topic to decrease the risk of severe infections, particularly due to gram-negative bacteria in cases with neutropenia.

## Acknowledgments

We would like to express our sincere thanks to Professor Bekir Kocazeybek, MD, and Pelin Yüksel, MD, PhD, for laboratory and technical support. We are grateful to Dana Clutter, MD, for her contributions and revision of the manuscript in terms of language. This study was financially supported by the İstanbul University Research and Projects Unit (Project no: 3847).

## Ethics

Ethics Committee Approval: İstanbul University Cerrahpaşa Faculty of Medicine Ethics Committee (approval number: 2009/22079) (15.07.2009); Informed Consent: It was taken.

## Figures and Tables

**Table 1:**
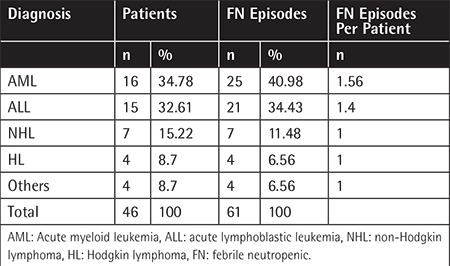
Hematological diagnoses of patients and number of febrile neutropenia episodes.

**Table 2:**
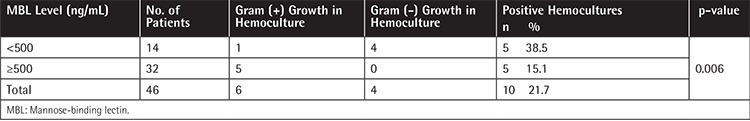
Rates and distribution of bacteremia according to mannose-binding lectin levels.

**Table 3:**
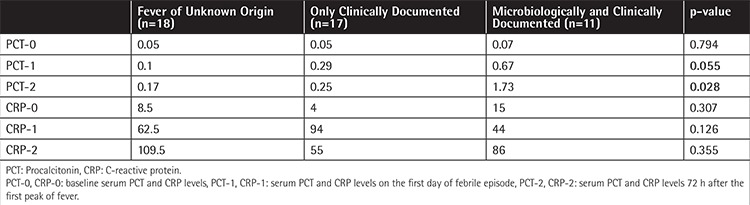
Median serum procalcitonin and C-reactive protein levels in three patient groups with febrile neutropenia.
